# Results from Poland’s 2022 Report Card on Physical Activity for Children and Youth

**DOI:** 10.3390/ijerph19074276

**Published:** 2022-04-02

**Authors:** Paweł Zembura, Agata Korcz, Hanna Nałęcz, Elżbieta Cieśla

**Affiliations:** 1 Department of Management, Organisation and Economy, Jozef Pilsudski University of Physical Education in Warsaw, 00-968 Warsaw, Poland; 2 Department of Didactics of Physical Activity, Poznan University of Physical Education, 61-871 Poznan, Poland; korcz@awf.poznan.pl; 3 Department of Child and Adolescent Health, Institute of Mother and Child, 01-211 Warsaw, Poland; hanna.nalecz@imid.med.pl; 4 Institute of Health Sciences, Medical College, Jan Kochanowski University of Kielce, 25-369 Kielce, Poland; eciesla@ujk.edu.pl

**Keywords:** physical fitness, sedentary behavior, Active Healthy Kids Global Alliance, physical activity promotion, Global Matrix, school, government, sport participation, organized physical activity, active play

## Abstract

This paper presents the methodology and results of Poland’s 2022 Report Card on physical activity (PA) of children and adolescents, as part of the Global Matrix (GM) 4.0 project. The aim of this paper is to discuss the current state of PA of children and adolescents in Poland. Grades were assigned to ten indicators of behaviors, physical fitness and sources of influence or settings, based on a synthesis of the best available data. In Poland two indicators: Overall Physical Activity (OPA) and Active Play were not assessed. Out of the other indicators, School received the highest score B+, whereas the other indicators had generally moderate to weak scores (between C+ and D). Scores for Organized Sport and Physical Activity, School and Physical Fitness indicators were improved compared with the previous GM, whereas scores for Active Transport and Government were lower. No positive changes regarding OPA or Sedentary Behaviors were observed. One of the limitations of PA promotion in Poland is that government-level PA policies are overly focused on organized PA and sport. Recommendations for improving PA monitoring in Poland, influencing PA behaviors and strengthening PA settings and sources of influence are also discussed.

## 1. Introduction

The evidence-based knowledge of the influence of physical activity (PA) on individual health [1], as well as an understanding of its influence on public health [2,3,4] has grown of late. There is emerging evidence suggesting that participation in physical activity may provide benefits beyond physical health [5]. Yet, among children and adolescent populations we are faced with a global epidemic of insufficient PA [6] and obesity [7], further exacerbated by the effects of the COVID-19 pandemic [8].

While the insufficient PA of children and adolescents is commonly considered a global issue, the actual programs to solve the problem primarily occur on a national level [9]. Efficient policies and programs require up-to-date, valid national-level data to define issues, measure progress, determine intervention tools and evaluate the outcomes of these interventions. In recent years a number of international research initiatives have been developed to gather data on a continual basis to improve understanding of PA behavior trends on the international level, which also provide a structure for analyzing PA trends on a national level [10,11].

One such initiative is the Active Healthy Kids Global Alliance (AHKGA) network, for which national expert teams comprehensively summarize the current state of PA on a national level in the form of Report Cards [10]. The data from national Report Cards has been compiled to form the Global Matrix (GM) of Physical Activity Report Cards. The first edition of GM launched in 2014 involved 15 countries while the upcoming 2022 release of GM 4.0 is expected to consist of 60 countries [12]. National Report Cards are conducted according to a harmonized and transparent Report Card development process, in which each country’s expert team compiles the available evidence from local, national, or international studies, as well as grey literature such as national reports or unpublished data, and later synthesizes findings and reaches consensus for the grading of each indicator [10].

The following indicators are the core of the analysis in the most recent GM 4.0: Overall Physical Activity (OPA), Organized Sport and Physical Activity, Active Play, Active Transportation, Sedentary Behaviors, Physical Fitness, Family and Peers, School, Community and Environment and Government. Each of the ten core indicators are defined and from one to six specific benchmarks describing every indicator are provided. Benchmarks serve as reference points for grade assignment, although not all have to be used. Based on available data, the research team decides upon a grade for each indicator from A+ (which refers to succeeding with between 94 and 100% of children) to F (succeeding with less than <20% of children). The research team might also decide to mark indicators as Incomplete (INC) in case of insufficient data. The grading system used when translating percentile values describing scores for each of the indicators to grades is provided. Next, draft grades and their rationale are submitted to members of the AHKGA Executive Committee who audit them to ensure consistency of scoring [10].

Generally, the country Report Cards facilitate global, as well as local efforts promoting engagement in PA. It enables the comparison of Report Card results from multiple countries to facilitate mutual learning and subsequently improve grades [10,13]. This network of diverse alliances (including countries in all socio-economic groups) allows for critical evaluation of different aspects of PA over time.

Since 2016, Poland has been involved in the AHKGA and became a part of the international GMs with the country Report Cards for 2016 (GM 2.0) and 2018 (GM 3.0). The results of the two previous Report Cards showed a highly insufficient PA among children and adolescents in Poland, and also revealed gaps in research, interpretation, and reporting of PA and SB in this population. Since the GM 3.0, the availability of data on PA in Poland has improved. Information about PA and sedentary behavior (SB) levels are collected in different age groups in representative samples of children and adolescents in projects such as Health Behavior in School-aged Children (HBSC) and the Childhood Obesity Surveillance Initiative (COSI). However, challenging the inactivity crisis is complex, and a single piece of research provides just partial information about the issue. Thus, projects summarizing the actual state of knowledge on a national level that further enable simplified comparisons between nations and can be translated into policies are of value.

In the most recent wave of research, GM 4.0, the expert team conducted a detailed analysis of national data sources for ten indicators, further detailed with benchmarks. The 2022 Active Healthy Kids Poland Report Card provides an up-to-date, evidence-based resource on PA and SB performance and opportunities in a wide context. The purpose of this paper is to summarize the process and results of the 2022 Report Card for Poland.

## 2. Materials and Methods

The goal of the development of the Report Card in Poland was to compile the best available published and unpublished evidence in order to assign letter grades to physical activity indicators based on harmonized benchmarks and grading rubrics [14]. A crucial quality of the GM is that the benchmarking process is in large parts synchronized between the countries and is based on a unified methodological background, such as defining indicators, as well as a common benchmarking process and grading system [10]. A general trend in the project is to improve reliability and the cross-country comparability of scores. However, the research teams in the countries participating in GM 4.0 still have the ability to decide upon multiple methodological issues that impact scores and grades [15]. As an example, they decide on the exact inclusion criteria of data sources that will later inform decisions on grades, or on a way to decide on a final grade based on various data sources. Decisions made by the Polish team are discussed in this section. In GM 4.0 minor changes have been made in comparison to the GM 3.0 regarding scoring procedures and benchmarks, such as providing a benchmark that might be used for qualitative analysis of PA policy, which is a part of a Government indicator in GM 4.0 [16].

In Poland the research team consisted of four researchers working for public research institutions. Several external experts and stakeholders were also involved at the different phases of the project. This was the third edition of the GM that the Polish team has participated in. The research process started in June 2021 and finished in January 2022.

In the analysis we took into consideration studies that used data gathered from 2018 to March 2020. However due to a lack of other sources it was decided to include sources of data gathered from 2016 onwards for Physical Fitness, Community and Environment and Governance indicators. To ensure representativeness, it was decided to include only data gathered from at least a regional (voivodeship) level (i.e., they should not refer just to a single city or community (gmina). In the case of Governance, we analyzed actions undertaken on a government-level or a national scale. In cases when more than a single dataset from a single research project (such as COSI or HBSC) was available, we analyzed only publications referring to the most recent iteration of the study. If there were several articles referring to the same dataset but which applied different procedures for data cleaning or extraction, we only used the articles using the original procedure.

A decision was made not to include studies or data gathered since the start of the national restrictions associated with COVID-19 pandemic (starting from March 2020). For the purpose of comparability with the previous editions of the GM in Poland and due to challenges of analyzing and combining studies conducted throughout COVID-19, it was decided to separate data from before and after the start of the pandemic. Thereby, this analysis can serve as a starting point for studying the impact of pandemic on PA.

For the purpose of identifying relevant data, the basic catalogue of keywords was developed based on previous experience in GM 3.0 and was then extended, using synonyms and country-specific wording. We sought scientific literature as well as grey literature (such as expert reports). PubMed, EBSCO and Google Scholar databases were used to search for scientific and grey literature, and later other web search engines were used to identify additional sources of information on the Internet and specifically, websites of public institutions conducting research related to PA. The searches were conducted in English and Polish.

A single researcher from the research team led the analysis regarding each indicator, and a separate analysis was initially conducted for every indicator. During the process, several stakeholders were consulted with the aim of identifying additional sources of information that would fit the set criteria. These stakeholders were previously identified by research team members as representatives of organizations involved in PA promotion and included: public servants, representatives of Non-Governmental Organizations (NGOs) and local authorities.

After obtaining a broad range of literature, a leading researcher on an indicator was tasked with the initial screening of the dataset, excluding duplications and data that would not refer directly to definition or benchmarks of an indicator, as defined in the GM 4.0 guidelines. At this stage other team members were invited to consult and add their suggestions to a list of sources gathered regarding the specific indicator.

For data sources assessment, we used a modified Downs and Black checklist for non-randomized studies [17]. The checklist has been limited to ten questions relevant for cross-sectional studies using the expert method. We assessed data sources rather than research articles or articles, meaning that if we had more information about the dataset than available in a particular article using data from this dataset, we would account for all the available information during the assessment. This was due to the observation that in two studies using the same dataset, various information regarding the dataset might be published, yet this does not affect the quality of the data.

Each data source was assessed by at least two research team members. In the case of a disparity of assessments by two researchers that exceeded two points, another researcher assessed the source and the extreme result was excluded from further analysis. An arithmetic mean of two marks was used for a data source assessment. Decisions on cut-off points were made separately for indicators describing behaviors and PF (higher cut-off) and sources of influence (lower cut-off), due to a lower availability of a high-quality data and other characteristics of benchmarks in reference to indicators discussing sources of influence. Sources regarding the Government indicator underwent a different process, as the assessment was based primarily on information and data published by the government, Supreme Audit Office reports and data gathered by the National Statistical Office.

To ensure improved comparability and consistency of the results in the next editions of GM, decisions were made by the Polish team that aimed to achieve quantifiable results on all indicators except for Government. This was particularly relevant for PA behaviors as the benchmarks were more consistent. To make decisions about grades, we used a cascade approach. Firstly, given the variety of age groups among children and adolescents (5–17) we decided to divide the analysis into four age groups, so that even in the case of not having much data on a particular age group, it would still be of the same importance as other age groups. Four age groups were distinguished: 0–4-year-olds, 5–9-year-olds, 10–14-year-olds, and 15–19-year-olds, which were based on the World Health Organization (WHO) and United Nations International Children’s Emergency Fund (UNICEF) classifications [1,18,19]. If studies that included data on just a single gender were included in the analysis, we would firstly conduct the analysis by gender in each age group. Secondly, if more than a single source was analyzed within the age groups, we adjusted the analysis based on sample size. After obtaining an average score per age group, we calculated an average score per benchmark. Next, we calculated the average result for an indicator by considering the results of the available benchmarks. The final grades for the indicators were given in accordance with the values defined by the GM 4.0 guidelines.

It should be noted that an exception has been made in reference to the Sedentary Behaviors indicator, for which a final score has been made based on the consensus of the Research Team. A quantifiable result was based on just a single screen time behavior—watching TV or movies. However, having information on the prevalence of the other screen time behaviors and given the changing trends in media consumption among children and adolescents, it was decided that use of a single proxy of watching TV and movies would lead to underestimation of SB in the report [20].

## 3. Results

The 2022 Report Card for Poland is the third such assessment of PA. Among the ten core indicators, the country scored three grade “Cs” and two grade “Cs−”. One indicator was graded as a “B+”, one as a “C+” and one as a “D”. “Incomplete” was assigned to two indicators (OPA and Active Play). The rationale for each grade is presented in Table 1.

## 4. Discussion

This study summarizes the process and results of the Poland’s 2022 Report Card, using the best and most recent data to evaluate progress in behaviors, PF as well as settings and sources of influence on PA for children and youth.

The GM grades on PA among children and adolescents provide an opportunity to describe and examine the global situation of PA in a comprehensive way. Poland joined the AHKGA and developed its first Report Card in 2016 [38], which was published as part of the GM 2.0 [13].

### 4.1. Availability of Data and Methodology

In terms of availability of data, in compared with GM 3.0, and in reference to OPA, Organized Sport and Physical Activity, Active Transport, Family and Peers and School, in GM 4.0 a more diverse dataset was available, covering a broader population of children. Importantly, in the GM 4.0 we accessed data on children younger than 11 years-of-age for the first time, which is important progress since the last 2018 Report Card. In reference to the School indicator, we were able to use more benchmarks, while lower availability of data was acknowledged regarding Family & Peers and Physical Fitness indicators. Both indicators require more focus from researchers, as virtually no up-to-date data was available. Out of the ten core indicators in the project, two indicators—Active Play and OPA, were not assessed.

Changes regarding methodology at international and national levels have to be considered when comparing the grades and to understand data availability. For the first time the research team merged results of multiple studies on a national level regarding each indicator. This, in turn, led to a single score that was directly transferable into a grade. This was not applied to Sedentary Behaviors due to the inability to calculate various screen time behaviors, and Government, as the indicator is not fully quantifiable. Importantly, the decision was taken that availability of data on OPA did not allow assessment of the indicator based on GM 4.0 benchmarks.

### 4.2. Discussion on GM 4.0 Grades in Poland

The highest grade was observed for the School indicator (B+). The remaining indicators (both PA related behaviors and settings/sources of influence) are generally moderate to weak (from C+ to D).

Compared to Poland’s last assessment of PA (see Table 2) for children and adolescents in 2018 [39], the grades of the three indicators in the 2022 Report Card slightly improved (Organized Sport and Physical Activity, Physical Fitness and School). Sedentary Behaviors (grade of D), Family and Peers (grade of C−) and Community and Environment (grade of C) remained unchanged from the previous assessment. Active Transportation (grade of C−) with Government (grade of C) were assessed slightly lower than in 2018. Again, Active Play was graded as incomplete (INC) due to the lack of available data. Moreover, in this edition also OPA was graded as incomplete (INC) due to methodological changes. However, as indicated, the score for the OPA indicator would decrease since GM 3.0 if we used the previous WHO recommendations as a benchmark [40]. In the analyzed studies a lower percentage of children and adolescents met the former WHO recommendations than in GM 3.0. In fact, our 2022 score could be more accurate as it referred to more than just a single study and covered a broader population of children and adolescents (based on age groups).

An improved score in reference to Organized Sport and Physical Activity might be partly associated with the increasing availability of sport programs generally and in the school environment. In Poland, PA is a domain of the ministry responsible for physical culture (as of March of 2022 this is the Ministry of Sport and Tourism) so PA promotion is viewed through the lens of sports [30]. In fact, current national programs to promote PA are mainly implemented through sport clubs and sport associations, which might lead to increasing opportunities to participate in organized sport. Yet, the recent development of the commercial sport and fitness sector in Poland is worth acknowledging as this might contribute to a better grade.

Based on active transport behaviors, the Active Transportation indicator received a lower score than in GM 3.0. In the previous Report Card data on children younger than 11 years-of-age were not available. According to the COSI study, younger children seem to actively travel to school less frequently [25]. Thus, adding data on younger age groups to the analysis could have reduced the score.

The Sedentary Behavior indicator was graded “D”, the same as in GM 3.0. An average score was estimated only for watching TV and movies for less than 2 h a day. However, considering the prevalence of the other screen-time behaviors of Polish children and adolescents [21,41], the decision was taken to keep the score lower so it would reflect these behaviors. It is clear that a large number of children and youth in Poland spend a lot of time in sedentary activities, yet summarizing screen time behaviors is a challenge.

The Physical Fitness indicator scored slightly higher than in GM 3.0, yet this time only a single regional study informed the grade [27]. Furthermore, the results of just four tests were analyzed. Therefore, the representativeness as well as breadth of data is limited.

The grade for School has been relatively stable since the first national Report Card [38]. It shows the crucial role played by the education system in PA in children and youth (with a grade of “B” in both 2016 and 2018). In the last two years, new or extended programs to increase PA in schools in Poland have been undertaken by the ministry responsible for physical culture by financing additional afterschool sport classes [30]. These programs aim to finance extracurricular sport classes for pupils (Szkolny Klub Sportowy (SKS)) via sport clubs and Physical Education (PE) teachers, or directly subsidize sport clubs (Klub). The Programs have increasing budgets and reach (SKS had 250 thousand participants in 2021) [30,42]. With a sufficient number of mandatory PE lessons (three or four depending on school level), high participation in those lessons and better school infrastructure, the School indicator seems to be generally improving.

Government support for PA promotion among school-aged children and youth received a lower grade than in GM 3.0. While programs undertaken during the 2016–2018 period are being continued and are expanding, little has changed in terms of the importance of PA on the political agenda [30]. At a government level, PA promotion is still focused on sporting activities, which proved to be insufficient based on PA trends in Poland. While the programs, strategies and policies seem solid, based on analysis of HEPA PAT v2 and the scoring rubric published by Ward et al. [16], these tools aim to measure procedures rather than the quality of actual actions. Such areas are strong in bureaucratic sport systems, which was recognized in Poland [43]. Thus, while the programs are precisely planned in terms of source of financing, responsibility and actions associated with particular goals, the results (outcomes and impact) and effectiveness of the government actions are not evaluated [9]. In particular, evaluation of some of the programs is only a formality which does not lead to knowledge generation. Other factors contributing to the lower score include: enduring low breadth of policies to promote PA, a governmental focus solely on sport and school environments, a lack of leadership regarding PA promotion (a new strategy to promote PA was not established in 2020 after the last plan expired) and ignoring SB as a challenge that might be hard to solve just through extracurricular PE or sport. Another contributing factor is that the ministry responsible for physical culture has not published online the results of any studies or reports that refer to PA since 2019 [44]. Prior to 2019, the ministerial website was a go-to-resource for local authorities while developing strategies aimed at PA and was regularly updated with valuable resources for stakeholders.

### 4.3. Methodological Limitations of GM 4.0 in Poland

Despite the slowly expanding breadth of data in Poland, its diversity is quite limited. As an example, all studies from which we used the data to assess PA behaviors were conducted by the same institution, the Institute of Mother and Child in Warsaw [22,23,25]. The methodology and sampling in most of the studies have consistently utilized similar procedures.

From the retrospective view of the three editions of GM in Poland we can observe that, other than the HBSC survey, there is no data gathered consistently on the PA of children in Poland. HBSC is a single source that has been repeatedly cited in GM reports in Poland throughout the three editions as it covers indicators such as OPA, Sport & Organized PA, Active Transportation and the Sedentary Behavior of adolescents between 11- to 15-years of age [38,39]. Yet, even in reference to the HBSC study a change to some non-obligatory parts of the survey has been made in the most recent 2018 edition in Poland, such as not analyzing adolescent perceptions of support to participate in PA (Family & Peers indicator), which affected data availability for this indicator in GM 4.0 [21]. This dependence on a single source of consistent data in Poland might be changing with the introduction of the COSI study since 2016 [20]. However, the data gathering process in the next edition has yet to be confirmed. Having access to just a single study focusing on adolescents limits opportunities to observe trends regarding PA.

As HBSC and COSI are the main data sources regarding PA behaviors in Poland, it has to be acknowledged that the exact question regarding meeting PA recommendations is not in line with the new WHO PA recommendations for children and youth (5–17) which focuses on an average of 60 min MVPA per day [1,22].

Furthermore, still no large-scale device-measured studies have been conducted in Poland. Although there have been some initial small-scale studies using devices to gather data on PA, these have not led to larger research projects that could be included in the GM 4.0 analysis. A need for device-measured PA is relevant to the OPA and SB indicators. Based on the limitations of assessing SB based on screen-time behaviors, it seems clear that consistent SB monitoring requires other research methods to decrease the impact on measurement of trends in particular screen time behaviors [21]. Further research might be needed to change the form of questions about SB; the lifestyle and use of media of today’s youth, especially the widespread use of tablets and smart phones, need to be considered. Nowadays digital technologies play a considerable role in young peoples’ lives (e.g., computers in education and in extracurricular activities and in leisure time). Therefore, it may be challenging to increase the proportion of children and adolescents who adhere the recommended limit of two hours of screen exposure per day.

The Lack of data to form a grade for the Active Play indicator was acknowledged. The Active Play indicator was also not included in the Report Cards analysis in 2016 and 2018. The incomplete grades for active play of children and youth reflect a lack of nationally representative data. Indeed, 29 of 49 countries were unable to find available data to grade this indicator in 2018 [10]. The recent COSI study reported only on how many hours per day in their free time that students are physically active. 13% of the 2nd grade (7–8 year-olds) were physically active in their free time for more than 2 h a day during the weekday and 38.1% during weekend days [25]. However, unorganized and organized activities were merged into a single question (e.g., spends time actively outdoors—for fun, playing, jogging, or attends indoor sports activities—sports halls, clubs). Therefore, evaluation of unstructured/unorganized active play was impossible. Active play might be a potentially useful tool of future strategies aimed at increasing PA among children and youth. Therefore, more attention should be paid to these areas and future surveys need to include a simple and well-constructed question to parents and/or young people to estimate the engagement in active play.

In reference to the Physical Fitness indicator, on a national level we were not able to access much data consistent with the benchmark. On the one hand, through the initiative of the Ministry of Sport and Tourism in 2016, a platform was developed to gather population data on PF of children and youth. In the several large-scale sport-for-all programs founded by the ministry, the input of participants’ PF test data was made mandatory for school clubs and trainers. Over two million children and adolescents’ physical fitness tests have been inputted into a database called the *National Talent Database* (Narodowa Baza Talentów) in 2017 alone [45]. Yet, the platform and the fitness tests used in it serve primarily for talent identification in sport, which is in line with the policy focusing on sporting success [46]. It is, however, not consistent with the concept of health-related PF as adopted by Tomkinson et al. [26] and used in the last two GM editions. The results of these studies are not published publicly, nor are summaries available. Various programs use different PF tests other than the ones used in Eurofit. As a result, the database is under-utilized for policymaking purposes of comparison and we were not able to use it within this project. There is also a PF observatory in Poland that gathers PF data in 10-year cycles, yet the 2020 edition of the study has not been launched.

## 5. Conclusions

The results presented in the paper suggest that the situation regarding PA and screen-time behaviors is particularly concerning. In contrast, school is an important setting in Poland for the promotion of PA, especially due to the high level of organized sports participation and the mandatory time devoted to PE in schools. A strong commitment from the government is required at all levels to drive a cultural shift that would see Polish children and youth be physically active every day.

### 5.1. Recommendations on Data Availability

On a general level in Poland, changes are required to ensure consistent data gathering processes regarding some of the indicators. Indicators such as Physical Fitness, Family and Peers and Active Play are not covered in the regularly conducted studies that would ensure consistency of data and trend observations.

The current monitoring of the PA of children and adolescents in Poland is based on studies that use former WHO recommendations regarding OPA (HBSC, COSI). A decision has to be made about if and how to ensure the comparability of Polish data to WHO recommendations. On the other hand, the strength of these studies lies in the consistency of questions asked. Yet, if WHO recommendations become a new standard for PA cut-off points, using inconsistent benchmarks will result in issues with comparability on an international level.

In comparison to some other countries in the region, Poland seems to be lagging behind in conducting studies using device-measured PA [47]. An increased focus on methods other than surveys is required to test reliability of the previous research. Data from device-measured studies would be relevant for the Sedentary Behaviors indicator, given the limitations of the current data.

In reference to the Physical Fitness indicator, we propose the adoption of a uniform strategy for determining the level of PF in school-age children with the use of Eurofit test battery in government-ordered studies and programs. Currently, despite having a very broad database of tests results from multiple nation-wide projects, it is inconsistent. Thus, data is not utilized for health or epidemiological purposes. The public availability of data has to be increased as well. We also observed that tests that assess children’s motor abilities are more popular than those related to health, i.e., circulatory and respiratory capacity, which needs to be rectified in future studies.

This is the third Polish Report Card and, yet again, it was not possible to assign a grade to the Active Play indicator. A more informed picture of children’s active play needs to be built.

### 5.2. Recommendations on Grades Improvements in Poland

Through the last GM editions in Poland, we highlighted persistent narrow breadth of policies aimed at improving the PA of children and adolescents [48]. With the ministry responsible for physical culture in charge of PA promotion, the focus is primarily on sport-related initiatives, whereas other contexts of PA promotion remain almost entirely unrecognized.

Thus, in our view, it is necessary to review the current PA promotion and its place in strategic documents. It is necessary to step beyond the narrow approach of sport as the solution to decreasing PA and introduce more efficient PA promotion. To ensure that PA is viewed in a broad sense, implementing national guidelines on SB might be of value. The responsible ministries should also review the importance of SB from a health and education perspective.

Government decisions apart, a nation-wide campaign is required to promote national and international recommendations regarding PA and the screen time of children and adolescents. Although national recommendations have been developed in Poland, these have not been promoted, and as a result might be not known beyond the scientific community. Attractive, up-to-date campaigns are needed to inform society and stakeholders about the benefits of PA and the dangers of SB.

Based on the GM results, schools in Poland might be considered as a base for PA promotion. Our suggestion is again to promote PA in the context of transportation to school, active breaks and programs that would lead to increasing the attractiveness of PA for children and adolescents (especially girls). Given the high level of autonomy and varied settings of schools’ soft policies, undertaking bottom-up plans for a “one-hour goal of PA” should be promoted.

In reference to organized PA and sport, as well school PA promotion, we suggest the quality of the programs introduced by the Ministry of Sport and Tourism and local governments should be examined more closely. With the increasing government budgets for these programs, they require policies targeting less active groups and also review of their effectiveness and impact [48]. Furthermore, the promised value of the elite sport sector for PA promotion must be reviewed as currently it is considered a given, despite its contribution remaining unclear.

## Figures and Tables

**Table 1:** Grades and rationales for Poland’s 2022 Report Card.

Indicator	Grade	Rationale
Overall Physical Activity	INC	A decision was taken to not grade the overall PA indicator in Poland. Physical activity monitoring system for children and adolescents in Poland primarily uses the Prochaska screening test, which does not give the opportunity to refer to the most recent WHO PA recommendation for children and adolescents—*% of children and youth who meet the Global Recommendations on Physical Activity for Health, which recommend that children and youth accumulate at least 60 min of moderate-to vigorous-intensity physical activity per day on average*, which is the main benchmark to assess PA in GM 4.0. No data using device-measured PA was available.However, if we analyze the most relevant studies based on the 7 days/60 min cut-off, a decreasing trend of OPA in comparison with the previous editions of the GM in Poland could be observed: 17.2% adolescents aged 11–15-years met the WHO guidelines for moderate-to-vigorous physical activity (MVPA) on the basis of HBSC 2017/18 [21]. In preschoolers, 19.3% of 3-year-olds, 18.5% of 4-year-olds, 15.8% of 5-year-olds, and 16.2% of 6-year-olds met the recommendations of 7 days/60 min MVPA on the basis of a pilot study conducted for the Ministry of Sport and Tourism [22]. 10% of 15-year-old girls met the 7 days/60 min MVPA recommendation [23]. 9.6% of 17-years-olds met the WHO guidelines of MVPA on the basis of study extending HBSC [24]. Based on the above data, a final score using previous WHO recommendations would lead to an average of 16.8% children and adolescents meeting recommendations. This corresponds to a grade F. However, as explained above, a decision was made to mark this indicator as incomplete (INC).
Organized Sport and Physical Activity	C+	In reference to a single benchmark *% of children and youth who participate in organized sport and/or physical activity programs*: 41.4% of adolescents aged 11–15-years participated in organized team sports, and 26.4% in organized individual sports on the basis of the HBSC study (52.3% of adolescents participating in any organized sport, by own calculations). 62.4% of the 2nd grade (7–8-year-olds) students, and 40.3% of 7th grade (12–13-year-olds) students participated in organized sport (COSI) [25]. The final score of 54% corresponds to a grade C+.
Active Play	INC	Active play was graded as incomplete (INC) due to inadequate information to assign a grade. Although national surveys (such as COSI) collected active play-related data, none of them reported on the *% of children and youth who engage in unstructured/unorganized active play at any intensity for more than 2 h a day*
Active Transportation	C−	For assessing the Active Transportation indicator, we used a single benchmark—*% of children and youth who use active transportation to get to and from places* (e.g., *school, park, mall, friend’s house)*. Two sources of data were used: 48.3% of adolescents aged 11–15-year walk or cycle to school according to HBSC (own calculations). 39.6% of 2nd grade students, and 55.8% of 7th grade students actively travel to school (COSI) [25]. Based on the results and sources of data, an average score of 44.8% was estimated, which corresponds to a grade C−.
Sedentary Behaviors	D	While grading Sedentary Behaviors we referred to a single benchmark *% of children and youth who meet the Canadian Sedentary Behavior Guidelines (5–17-year-olds: no more than 2 h of recreational screen time per day)*: 73.9% of 2nd grade pupils spent less than 2 h a day in front of the TV screen and using electronic media on school days, while 43.3% did on weekend days, according to their parents or guardians in the COSI study [25]. 38.6% of the 7th grade students stated watching TV or movies for less than 2 h/day on school days while 17.3% did on weekend days (COSI). Further, 38.6% of 7th grade students declared spending less than two hours a day in front of a computer screen, tablet or smartphone on school days, while 24.5% did so on weekends (COSI). 39.2% of adolescents aged 11–15-years spent less than 2 h watching movies and programs in their spare time during school days, while 17.4% did during weekend days (HBSC) [21] 66.3% spent less than 2 h playing games on a computer, console, tablet or smartphone or other equipment (other than movement games) on weekdays, while 43.7% did so on weekend days (HBSC). 29.3% used a computer, tablet or smartphone for other reasons in their spare time on weekdays, while 35.5% did so during weekend days (HBSC). 75.5% of 17-year-old high school students spent less than 2 h of their leisure time playing games on a computer, console, smartphone or other equipment (excluding games requiring activity) per weekday (HBSC extension) [24]. 19.7% used a computer, tablet or smartphone for other reasons in their spare time on weekdays for less than 2 h per weekday (HBSC study extension). An average score of 42.8% was estimated only for watching TV and movies for less than 2 h a day. However, given the prevalence of the other screen time behaviors in Poland it was decided by the consensus of researchers to grade the indicator D.
Physical Fitness	C	A single benchmark—*average percentile achieved on certain physical fitness indicators based on the normative values published by Tomkinson et. al*. [26] was used to grade Physical Fitness. The assessment was based on a single source of data. It included mean values of four (motor) tests assessing components of physical fitness: standing broad-jump, sit-ups in 30 s, handgrip test and bent-arm hang (9–11 year-old boys, 9–17 year-old girls), counted separately in each age and gender group. The mean percentiles for the long jump in relation to published international standards were similar in both gender groups (girls: 41.8%, boys: 40.4%) [27]. The mean percentiles for the handgrip test were slightly higher in the girls group than in the boys group at 40.5% and 38.2%, respectively [27]. In the sit-ups test, the average (mean) percentiles amounted to 66.3% in girls and 50.8% in boys. In the bent-arms hang test the analyzed, counted values were: 76.1% in girls and 67.9% in boys. Based on these results, we obtained an average score of 50.7%, which corresponds to a grade C.
Family and Peers	C−	The grade for family and peers was based on a single benchmark: *the % of family members* (e.g., *parents, guardians) who are physically active with their kids*. 48.0% of 2nd grade students and 32.9% of 7th grade students declared participation in PA with their family (parent/guardian, siblings) at a frequency from 1–2 days a week to everyday on the basis of the COSI study [25]. The average score was 40.4%, which is C−.
School	B+	The grade assigned to the school environment was based on three benchmarks: *(1) the percentage of schools where the majority (≥80%) of students are offered the mandated amount of PE (for the given state/territory/region/country).* 91.5% of the 2nd grade (n = 162) and 82.7% (n = 62) of the 7th grade classes realized a compulsory weekly number of physical education classes (COSI) [25]. *(2) the percentage of schools that offer physical activity opportunities (excluding PE) to the majority (>80%) of their students.* Extracurricular sports activities available to students at two levels of education were organized in 66.7% of schools (90 schools) (COSI). *(3) the percentage of schools with students who have regular access to facilities and equipment that support physical activity* (e.g., *gymnasium, outdoor playgrounds, sport in fields, multi-purpose space for PA, equipment in good condition).* 87% of school students had access to open-air sports outside school lessons, whereas indoor gyms are slightly less accessible to pupils—69.6% of schools declared that they were available to students (COSI). The average score was 77.4%, which corresponds to B+.
Community and Environment	C	To assess the indicator, we used four benchmarks covered by two sources of data. *(1) % of children or parents who report having facilities, programs, parks and playgrounds available to them in their community benchmark)* 68% of Polish citizens 15+ completely agree or tend to agree that the area where they live offers many opportunities to be physically active [28] 63% completely agree or tend to agree that sport clubs and other local providers offer many opportunities to be physically active, according to the Eurobarometer 2018 study [28] *(2) Percentage of children or parents who perceive their community/ municipality is doing a good job at promoting PA* (e.g., *variety, location, cost, quality)* 33% totally disagree or tend to disagree with a statement that their local authority does not do enough in relation to PA–Eurobarometer 2018 study. *(3) Percentage of communities/municipalities that report they have policies promoting PA benchmark* 35% of gminas (districts) reported having a current development strategy, and out of those gminas, 95% have goals related to physical culture. Therefore, 33.4% communities with development strategy with goals related to physical culture based on a report prepared for the Ministry of Sport [29]. *(4) Percentage of communities/municipalities that report they have infrastructure* ( e.g., *sidewalks, trails, paths, bike lanes) specifically geared toward promoting PA* 90% of gminas declared having school fields while 76% have sport halls next to the school in their community [29]. The final score of 50.9% corresponds to grade C.
Government	C	Three benchmarks were used to assess the Government indicator. *(1) Evidence of leadership and commitment in providing PA opportunities for all children and youth* In Poland at a governmental level, the topic of PA is primarily addressed through sports by the ministry responsible for physical culture. The largest programs promoted by the Ministry of Sport and Tourism are simple, uniform interventions to support access to sport for children and youth through sport clubs (Klub) and school trainers (Szkolny Klub Sportowy). Despite their large scale, there is little evidence that those programs affect PA levels of children and youth [30]. There is very limited reference to PA promotion in children and youth as part of governmental strategies other than through the lenses of sport and physical education. SB are not considered a separate challenge. There are no national policies aiming to impact SB other than through sport or PE programs [31,32]. *(2) Allocated funds and resources for the implementation of PA promotion strategies and initiatives for all children and youth. Demonstrated progress through the key stages of public policy making* (i.e., *policy agenda, policy formation, policy implementation, policy evaluation and decisions about the future).* In reference to funds and resources allocated to the implementation of PA promotion strategies on a national level, consistent progress can be observed in the amount of money spent on sport promotion programs aimed at children and youth through the channels directed by the ministry responsible for physical culture [30]. Subsidies for physical culture given by local authorities slowly increased between 2011 and 2016, whereas the trend in total spending from 2011 to 16 was not clear [33]. Despite the observed lack of success in achieving levels of PA levels in youth aimed for in the Sport Development Programme, the approach to promote PA through sports in Poland still continues [30]. Furthermore, in 2020, an operational plan in the area of PA promotion in Poland was not developed, despite being included in the plan (the previous Sport Development Programme expired in 2020) [32]. PA exists in some mid-term national strategies, yet these plans are fragmentary. On the other hand, in 2017 the very first national recommendations for health-enhancing PA in Poland were introduced. These include recommendations for four age groups of children and youth [34]. *(3) HEPA PAT v2 and the scoring rubric published by Ward et al.* [16] An analysis of governmental strategies, action plans and programs (as defined in HEPA PAT v2) referring to the PA of children and youth was conducted based on a modified methodology proposed by Ward et al. [16]. In comparison to Ward et al., in our analysis we decided not to use the number of relevant policies as assessment criteria but gave policy breadth (number of sectors) relatively more importance. The aim of the analysis was to assess the quality of policies aiming to affect the PA of children and youth. Four general mid-term national strategies included references to PA promotion. In addition, two specific topical strategies and five programs (HEPA PAT v2 definition) were identified [35]. One specific strategy aimed at PA promotion in Poland is the Sport Development Programme 2020 that is supported by Implementation Plan [32,36,37]. The total score for nine documents is 86.5 points (100 maximum). The breadth of documents is low as they refer to a limited number of policy domains: sport, environment, education and transport. All documents have specifications to promote PA, and a specific organization is identified as responsible for delivery of actions. Most (82%) have explicit reporting systems with a set frequency and format of reports, 91% have explicit references to funding to support identified actions, and 55% have explicit references to monitoring and evaluation of progress and impact of the policy. Taking into account available data it was decided by the consensus of researchers to grade the indicator C.

Note: Grades for each indicator were based on the percentage of children and youth meeting a defined benchmark: A+ is 94 to 100%, A is 87 to 93%, A− is 80 to 86%, B+ is 74 to 79%, B is 67 to 73%, B− is 60 to 66%, C+ is 54 to 59%, C is 47 to 53%, C− is 40 to 46%, D+ is 34 to 39%, D is 27–33%, D− is 20 to 26%, F is <20%, and INC is incomplete/insufficient data.

**Table 2:** Average grades by indicator and evolution of the grades from 2016 to 2022.

Indicators	2016	2018	2022
Overall Physical Activity	D	D−	INC
Organized Sport and Physical Activity	C	D	C+
Active Play	INC	INC	INC
Active Transportation	C	C	C−
Sedentary Behaviors	D	D	D
Physical Fitness	-	C−	C
Family and Peers	C	C−	C−
School	B	B	B+
Community and Environment	C	C	C
Government	C	C+	C

Note: INC = incomplete grade.

## Data Availability

Not applicable.
